# Multimorbidity: Not Just for the West

**DOI:** 10.5334/gh.835

**Published:** 2020-07-02

**Authors:** Amitava Banerjee, John Hurst, Edward Fottrell, J. Jaime Miranda

**Affiliations:** 1 Institute of Health Informatics, University College London, London, UK; 2 UCL Respiratory, University College London, London, UK; 3 Institute for Global Health, University College London, London, UK; 4 CRONICAS Center of Excellence in Chronic Diseases, Universidad Peruana Cayetano Heredia, Lima, PE

**Keywords:** global health, non-communicable disease, multimorbidity

## Abstract

“A multimorbidity lens creates exciting opportunities to reconceptualise health and wellbeing in all its complexity. We need to improve health metrics to capture this complexity and strengthen health services to respond to it.”

The ‘diseases of affluence’ model was turned upside down in the 1990s by the recognition that the burden of cardiovascular disease, diabetes, chronic respiratory disease, cancer and other chronic non-communicable diseases (NCDs) was not just high in high-income countries (HICs); 80% of the global NCD burden was in low- and middle-income countries (LMICs) [1]. This neglect of NCDs has delayed prioritisation and allocation of resources (human, financial and infrastructural) to biomedical research, training of health professionals and healthcare [2], leaving the poorest countries with the greatest need with the greatest gaps in care and in data.

Today, multimorbidity faces the problem single NCDs faced three decades ago: a global problem but barely recognised where it is likely to be most prevalent, and least researched in the settings where least is known. Multimorbidity, the ‘existence of multiple medical conditions in a single individual’ [3], has the person, not a disease, as the focus, but is still a comparatively underused term in literature and practice, with variable definitions. As with NCDs, the gap between multimorbidity burden and level of awareness, prevention and treatment necessary to avert the rising human, financial and healthcare costs in all countries is increasingly recognised, particularly in LMICs. Multimorbidity is not just for the West. In this sense, LMICs and their health systems have unique opportunities to leapfrog advances in healthcare delivery by not replicating the same structures of healthcare established in HICs. We explore this on three fronts, data, human resources, and care guidelines, whilst acknowledging progress (how to) in each aspect requires multisectoral support.

First, data for monitoring and accountability are required, since multimorbidity-related data remain limited [3]. Rather than generating disease-specific data, LMICs (and HICs) can move forward to generate a wider multimorbidity-based picture, in order to understand the most common clusters of multimorbidity and mainly those linked to higher morbidity [4], i.e. quality of life and patient burden [5], and mortality, based on specific disease burdens in certain geographical regions. The advantage of this approach would not only be in the understanding of conditions, but also in forming the basis of the design of the most promising interventions for research and implementation, together with the evaluation of patient-important outcomes [5,6].

Second, training of human resources. Improving services alone will not suffice without adequate training of the next generation of the health workforce. The existing crisis of human resources for health worldwide signals to the ‘elephant in the room’: we cannot afford to train health professionals into single-disease specialists [7]. This does not mean that specialists are not needed. On the contrary, we need generalists and specialists to better organise care such that specialist advice is available to greater numbers of people in community settings. This prioritisation does also provide further support to the call for universal health coverage [8].

Third, in terms of care, NICE has been at the forefront of producing guidelines for the management of multimorbidity [9]. The NICE multimorbidity guideline covers clinical assessment and management, and makes recommendations for research around organisation of care, holistic community assessment, polypharmacy and de-prescribing, and better prediction of life expectancy. This was developed in the UK context and to what degree this guidance is applicable, in part or in full, to LMICs remains to be explored and will vary by setting. So far, with few exceptions, most notably around HIV services [10], disease-centred and fragmented health services are more common in LMICs.

Whilst there may be many considerations beyond data, human resources and disease guidelines, LMICs should adapt sooner, faster and better to accommodate responses to multimorbidity rather than disease-specific structures. This is consistent with strengthening primary health care and universal health coverage via its contribution to high-quality healthcare [8]. These changes would be timely as they address conditions across the range of physical and mental non-communicable and communicable diseases [3,4], particularly as evidence grows to show that multimorbidity increases mortality in coronavirus (COVID-19) infection [11].

Indeed, multimorbidity creates an opportunity to re-conceptualise health and the lived experience of health and multiple conditions in ways that are likely to differ substantially from clinical ideas and medicalised views of wellbeing. Disease-focused, medicalised concepts of health sometimes neglect the complex social, financial and labour burdens of living with multiple chronic health conditions. Disease-specific actions vary, ranging from physical rehabilitation to timely medication adherence to modifying behaviours in terms of diet and physical activity, and emphasis is often placed on self-management and self-monitoring. But the complexities of actions and concepts of individuality and self are incompatible with the home and community environment where living with and managing multimorbidity takes place. Measures of multimorbidity need to describe wellbeing in this social context and strategies of community engagement and mobilisation are needed to ready and empower, not just individuals, but whole communities for coping with multimorbidity. Models of community mobilisation have shown promise in relation to a number of health outcomes in LMICs, and are likely to be particularly effective when combined with strengthening of data systems, and supply-side provision of quality, integrated care.

Addressing multimorbidity will be a challenge, a major one but necessary and long overdue. As our populations survive longer with multiple disease burdens over their lifetimes, more humane, high-quality, and long-term interactions with health services are required. Multimorbidity is the litmus test for the future of health and the experience of health, healthcare, and above all, the wellbeing of everybody, not only patients and caregivers, but also providers. There have been multiple attempts to better bridge the gap between simple intervention trials and the reality and complexity of implementation in real-world settings, e.g. the UK Medical Research Council complex intervention framework [12]. In health informatics, the prominence of continuously collected and utilised electronic health data in ‘science’, ‘evidence’ and ‘care’ is growing in the ‘learning health system’ model. The lens of multimorbidity may represent the best way of integrating various approaches across research, practice and implementation.

## Transparency Statement

The corresponding author affirms that the manuscript is an honest, accurate, and transparent account of the study being reported; no important aspects of the study have been omitted.

## References

[1] Murray CJ, Lopez AD. Mortality by cause for eight regions of the world: Global Burden of Disease Study. Lancet. 1997; 349: 1269–76. DOI: 10.1016/S0140-6736(96)07493-4 9142060

[2] Hurst JR, Dickhaus J, Maulik PK, et al. Global Alliance for Chronic Disease researchers’ statement on multimorbidity. Lancet Glob Health. 2018; 6: e1270–1. DOI: 10.1016/S2214-109X(18)30391-7 30420026

[3] Academy of Medical Sciences. Multimorbidity: A priority for global health research London: Academy of Medical Sciences, 2018 https://acmedsci.ac.uk/policy/policy-projects/multimorbidity.

[4] Whitty CJM, Watt FM. Map clusters of diseases to tackle multimorbidity. Nature. 2020; 579(7800): 494–496. https://www.nature.com/articles/d41586-020-00837-4 DOI: 10.1038/d41586-020-00837-4 32210388

[5] Montori VM, Gandhi GY, Guyatt GH. Patient-important outcomes in diabetes--time for consensus. Lancet. 2007; 370: 1104–6. DOI: 10.1016/S0140-6736(07)61489-5 17905147

[6] Dobler CC, Harb N, Maguire CA, Armour CL, Coleman C, Murad MH. Treatment burden should be included in clinical practice guidelines. BMJ. 2018; 363: k4065 DOI: 10.1136/bmj.k4065 30314981

[7] World Health Organization. Global strategy on human resources for health: workforce 2030 Geneva: World Health Organization; 2016 https://apps.who.int/iris/bitstream/10665/250368/1/9789241511131-eng.pdf?ua=1.

[8] Accelerating progress towards Universal Health Coverage. UHC2030. https://www.uhc2030.org/ (Accessed 19 October 2019).

[9] National Institute for Health and Care Excellence (NICE). Multimorbidity: clinical assessment and management. NICE guideline [NG56]. NICE. https://www.nice.org.uk/guidance/ng56 (accessed 15 September 2017).

[10] Mounier-Jack S, Mayhew SH, Mays N. Integrated care: learning between high-income, and low- and middle-income country health systems. Health Policy Plan. 2017; 32: iv6–12. DOI: 10.1093/heapol/czx039 29194541 PMC5886259

[11] Guan WJ, Liang WH, Zhao Y, Liang HR, Chen ZS, Li YM, Liu XQ, Chen RC, Tang CL, Wang T, Ou CQ, Li L, Chen PY, Sang L, Wang W, Li JF, Li CC, Ou LM, Cheng B, Xiong S, Ni ZY, Xiang J, Hu Y, Liu L, Shan H, Lei CL, Peng YX, Wei L, Liu Y, Hu YH, Peng P, Wang JM, Liu JY, Chen Z, Li G, Zheng ZJ, Qiu SQ, Luo J, Ye CJ, Zhu SY, Cheng LL, Ye F, Li SY, Zheng JP, Zhang NF, Zhong NS, He JX, China Medical Treatment Expert Group for Covid-19. Comorbidity and its impact on 1590 patients with Covid-19 in China: A Nationwide Analysis. European Respiratory Journal. 2020 DOI: 10.1183/13993003.00547-2020 PMC7098485 32217650

[12] Moore GF, Audrey S, Barker M, et al. Process evaluation of complex interventions: Medical Research Council guidance. BMJ. 2015; 350: h1258 DOI: 10.1136/bmj.h1258 25791983 PMC4366184

